# Long-range seed dispersal enables almost stationary patterns in a model for dryland vegetation

**DOI:** 10.1007/s00285-022-01852-x

**Published:** 2022-12-17

**Authors:** L. Eigentler, J. A. Sherratt

**Affiliations:** 1 Division of Molecular Microbiology, School of Life Sciences, University of Dundee, Dundee, DD1 5EH UK; 2 Mathematics, School of Science and Engineering, University of Dundee, Dundee, DD1 4HN UK; 3 Maxwell Institute for Mathematical Sciences, Department of Mathematics, Heriot-Watt University, Edinburgh, EH14 4AS UK

**Keywords:** Vegetation patterns, Periodic travelling waves, Wavetrains, Matched asymptotics, Nonlocal dispersal, Perturbation theory, 92B99, 92C15, 35B60, 35Q80

## Abstract

Spatiotemporal patterns of vegetation are a ubiquitous feature of semi-arid ecosystems. On sloped terrain, vegetation patterns occur as stripes perpendicular to the contours. Field studies report contrasting long-term dynamics between different observation sites; some observe slow uphill migration of vegetation bands while some report stationary patterns. In this paper, we show that long-range seed dispersal provides a mechanism that enables the occurrence of both migrating and stationary patterns. We utilise a nonlocal PDE model in which seed dispersal is accounted for by a convolution term. The model represents vegetation patterns as periodic travelling waves and numerical continuation shows that both migrating and almost stationary patterns are stable if seed dispersal distances are sufficiently large. We use a perturbation theory approach to obtain analytical confirmation of the existence of almost stationary patterned solutions and provide a biological interpretation of the phenomenon.

## Introduction

Patterned vegetation is a characteristic feature of semi-arid ecosystems, with vegetated regions alternating with bare ground on scales of tens to hundreds of metres (Gandhi et al. 2019). Such patterns are important both because of their direct effect on ecosystem function, and because they provide an early warning sign of catastrophic desertification (Elsen et al. 2020; Kéfi et al. 2014; Rietkerk et al. 2021). Mathematical modelling has been used extensively to better understand these patterns (Gandhi et al. 2019; Merchant and Nagata 2015; Sun et al. 2022); moreover, models have helped to highlight the presence of closely related patterning processes in a variety of very different ecosystems, including mussel beds, salt marshes and peat bogs (Martinez-Garcia et al. 2022; Rietkerk et al. 2004; Sherratt 2013a; Zhao et al. 2019).

On (gentle) slopes, semi-arid vegetation patterns usually consist of stripes running parallel to the contours (Valentin et al. 1999). In many cases, careful empirical observation shows that the patterns move slowly uphill, taking many decades to move one wavelength [Valentin et al. (1999, Table 5) and Tongway and Ludwig (2001)]. There is a simple intuitive explanation for such movement: rainwater flows downhill, so that the uphill edge of a vegetation stripe is relatively wet while the downhill edge is much drier. Consequently, plants tend to die preferentially at the downhill edge, and seeds tend to germinate preferentially near the uphill edge. This leads to uphill movement of the stripe on the time scale of the plant generation time. In many cases, field observations reveal differences between the uphill and downhill edges of vegetation stripes that are entirely consistent with this intuitive argument (Montaña et al. 2001; Tongway and Ludwig 2001).

However, there is also strong empirical evidence that some striped vegetation patterns on hillslopes are stationary (Dunkerley and Brown 2002; Mabbutt and Fanning 1987; Tongway and Ludwig 2001). More recently, Deblauwe et al. (2012) made a comparison of modern satellite images and declassified spy satellite images from the 1960s and 1970s, showing clear evidence of uphill migration in three of the six cases considered, but no evidence in the other three cases.

Mathematical models typically predict pattern migration on slopes, as a consequence of the downhill flow of water. Therefore, the observation of apparently stationary vegetation patterns is in apparent contradiction to models. Two main arguments have been proposed to resolve this contradiction. (1) Changes in soil structure: the regions between vegetation stripes are bare, and subject to significant surface erosion in run-off. This will tend to lead to hard dense soils, for which the equations of mathematical models may not be appropriate (Dunkerley and Brown 2002). (2) Seed dispersal in run-off: the downhill flow of rainwater may lead to a “secondary seed dispersal” that will counteract the tendency of stripes to move uphill (Saco et al. 2007; Thompson and Katul 2009). This was investigated in detail by Thompson and Katul (2009) who showed that it can indeed lead to stationary patterns, but only when the rates of downhill water flow and secondary seed dispersal are finely balanced.

The widespread evidence of stationary vegetation patterns suggests that neither of these proposed explanations gives the whole story. In this paper, we show that long-range seed dispersal (without a directional bias) provides an alternative mechanism for stationary patterns. Moreover, this potential mechanism is significantly more robust than those discussed above.

## Klausmeier model with long-range seed dispersal

### Klausmeier model

Semi-arid vegetation tends to exhibit spatial patterns because of the presence of short range activation and long range inhibition (Rietkerk and van de Koppel 2008), the combination of which is a standard mechanism for patterning (Meron 2015; Murray 2011). The inhibition arises from competition for water, which is long range because of rapid transport of water both within the soil and on its surface. Short range activation occurs because the presence of plants increases the infiltration of rain water into the soil, due to both the break-up of soil by the roots and the presence of organic matter on the soil surface: this makes it advantageous for plants to cluster together (Meron 2012; Ursino 2005; Valentin et al. 1999). A second local activation mechanism also occurs in many species: as plants grow, their root network spreads laterally, increasing water availability (Gilad et al. 2007; Meron 2012). Amongst the variety of mathematical models that have been proposed for these mechanisms, that of Klausmeier (1999) has been most widely used in recent years. It consists of coupled PDEs for plant density *u*(*x*, *t*) and water density *w*(*x*, *t*), where *t* is time and *x* is a spatial coordinate in the uphill direction:

1a $$\begin{aligned} \frac{\partial w }{\partial t}&= \overbrace{a}^{\text {rainfall}} - \overbrace{w}^{\text {evaporation}} - \overbrace{u^2w}^{\begin{array}{c} \text {water uptake} \\ \text {by plants} \end{array}} + \overbrace{\nu \frac{\partial w}{\partial x}}^{\begin{array}{c} \text {water flow}\\ \text {downhill} \end{array}} , \end{aligned}$$

1b $$\begin{aligned} \frac{\partial u }{\partial t}&= \underbrace{u^2w}_{\text {plant growth}} - \underbrace{bu}_{\text {plant loss}} + \underbrace{\frac{\partial ^2 u}{\partial x^2}}_{\text {plant dispersal}}. \end{aligned}$$

This is a dimensionless form of the model: see Klausmeier (1999) for details of the dimensional model and the rescalings used to obtain (1). The short range self-activation of plant growth is reflected in (1) by the assumption that the per capita rate of water uptake is proportional to plant biomass. The plant growth rate is also assumed to be proportional to water uptake on the basis that water is the limiting resource; however it should be noted that in some semi-arid regions nitrogen availability can also limit plant growth (Hooper and Johnson 1999; Stewart et al. 2014). Plant loss is assumed to have a simple linear form. Some recent models have included soil toxicity, which can arise via the decay of dead plant material, showing that this can play a significant role in vegetation pattern formation (Carteni et al. 2012; Iuorio and Veerman 2021; Marasco et al. 2014); however this is excluded from (1). Plant dispersal is represented by linear diffusion: this simplification is made for mathematical convenience, and the alternative use of a nonlocal dispersal term will be a key ingredient of the present work. The (dimensionless) parameter $a$ is proportional to mean annual rainfall. The use of a constant rainfall rate is a major simplification, since in most semi-arid regions rainfall occurs principally at certain times of year, and then only in relatively brief storms (Caylor et al. 2014; Istanbulluoglu and Bras 2006). Both of these complications have been considered in previous modelling studies (Eigentler and Sherratt 2020a, b; Guttal and Jayaprakash 2007; Kletter et al. 2009; Siteur et al. 2014; Ursino and Contarini 2006; Vezzoli et al. 2008). The parameter $b$ reflects both natural plant loss and the effects of herbivory. As well as grazing by wild and domestic animals, “herbivory” of woody vegetation includes human removal of trees for fuel, which has a significant effect on vegetation dynamics in many semi-arid regions (Berg and Dunkerley 2004; Dembélé et al. 2006; Hejcmanová et al. 2010). The parameter $\nu$ measures slope gradient. Some more recent models use representations of downhill water flow that are more detailed than the simple advection term in (1); in particular Gilad et al. (2004, footnote 18) derive a representation of surface water flow using shallow water theory.

Some authors have added a water diffusion term to (1) (e.g. Siteur et al. (2014) and Ursino (2005)), but we omit this in the interests of simplicity; the extension of our results to such an augmented model is a natural target for future work. A final simplification made in (1) is that all the parameters are homogeneous in space. We will retain this assumption throughout this paper, but it should be noted that some modelling research points to the potential importance of parameter heterogeneity in models for semi-arid vegetation, in particular its ability to increase resilience to reductions in rainfall (Bonachela et al. 2015; Yizhaq et al. 2014).

When the rainfall parameter is low ($a<2b$), the only spatially uniform steady state of (1) is the plant-free state $u=0$, $w=a$. Intuitively, the rainfall level is too low to support uniform vegetation. For $a>2b$ there are two vegetated uniform steady states. One is unstable to homogeneous perturbations. The other is stable to homogeneous perturbations but becomes unstable to inhomogeneous perturbations for sufficiently large values of $\nu$, via a Turing-Hopf bifurcation. Spatial patterns result, and these move in the positive *x* direction (uphill). Mathematically, this movement is most easily understood in terms of the dispersion relation, whose solutions are always complex-valued as a result of the advection term (Perumpanani et al. 1995; Sherratt 2005).

The most slowly moving of the patterns are unstable as solutions of (1), at least for the large values of the slope parameter $\nu$ that are ecologically realistic (Sherratt 2013a). Therefore, any pattern observed in solutions of (1) must necessarily move uphill at a speed that would be detectable in empirical studies such as comparison of satellite images taken several decades apart. As discussed above, only some of the available field data conforms to this aspect of the solutions of (1); other data reveals stationary patterns.

**Fig. 1:**
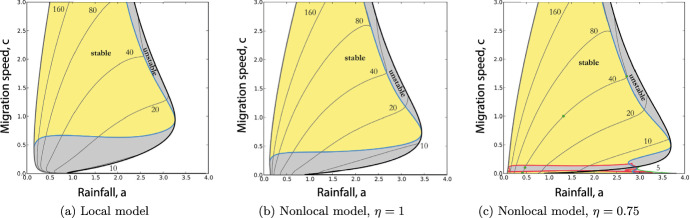
Existence and stability of patterns. A classification of the $a$-*c* parameter plane into regions of stable patterns (yellow), unstable patterns (grey), and no pattern existence (white) is shown for the model with local plant dispersal (**a**) and nonlocal plan dispersal (**b**, **c**). Note the thin region of stable solutions along the *a* axis in (**c**). The pattern existence region is bounded by a homoclinic orbit at low $a$ and by a Hopf bifurcation at higher $a$. Stability boundaries are either of Eckhaus (sideband) type (blue) or Hopf type (red); for full details see Bennett and Sherratt (2018). The annotated grey curves show a selection of wavelength contours. The parameters are $d= 0.5625, \eta = 0.75, \nu = 182.5, b= 0.45$, and $L = 7.24$. The figure is adapted from Bennett and Sherratt (2018) (color figure online)

Figure 1a illustrates the existence and stability of pattern solutions of (1), via shading in an $a$–*c* plane; here *c* is the uphill migration speed of the patterns. The key message from this figure is that stable patterns only exist when the wavespeed *c* is sufficiently large.

### A non-local model

Almost all models for patterned vegetation, including (1), assume local dispersal of plants via a diffusion term, representing the accumulated effect of seed dispersal and lateral vegetative growth through the continuum limit of a random walk. This is a convenient mathematical simplification, but is rarely accurate due to the occurrence of long range seed dispersal events. The distance a seed can travel from its source is influenced by external factors such as wind, as well as species specific characteristics, e.g. height of plant, seed weight—some plant species can even disperse seeds ballistically (Bullock et al. 2017). Secondary dispersal via animal or water transport can also affect the distance a seed can travel from its source (Neubert et al. 1995). A more realistic representation of plant dispersal is given by using an integral term based on a “dispersal kernel”, which is a probability density function describing the distribution of distances travelled by seeds originating from a single parent (Bullock et al. 2017; Nathan et al. 2012). This approach has been used previously in the context of semi-arid vegetation by Bennett and Sherratt (2018), Eigentler and Sherratt (2018) and Pueyo et al. (2008); our work builds directly on this foundation.

Replacing the diffusion term by a nonlocal dispersal term in (1) gives

2a $$\begin{aligned} \frac{\partial w}{\partial t}&= a-w-u^2w + \nu \frac{\partial w}{\partial x}, \end{aligned}$$

2b $$\begin{aligned} \frac{\partial u}{\partial t}&= u^2w - bu + d\left( \int _{-\infty }^\infty \phi (x-y)u(y,t) {{\text {d}}}y - u(x,t)\right) , \end{aligned}$$

where $\phi (\cdot )$ is the dispersal kernel and satisfies $\int _{-\infty }^{\infty }\phi (\xi )\,d\xi =1$. The choice of dispersal kernel depends on the plant species, and there is an extensive literature on this topic (see Nathan et al. (2012) or Bullock et al. (2017) for review). From a mathematical point of view, the “Laplace” kernel function

3 $$\begin{aligned} \phi (\xi )=\frac{1}{2}\eta \,e^{-\eta |\xi |}, \quad \eta >0, \end{aligned}$$

enables significant simplification, and throughout this paper we will restrict ourselves to this kernel. Equations (2) can then be reduced to a local PDE by writing

$$\begin{aligned} j(x,t)= \int _{-\infty }^\infty \phi (x-y)u(y,t) {{\text {d}}}y, \end{aligned}$$

and noting that

$$\begin{aligned} \frac{\partial ^2 j}{\partial x^2}+\eta ^2(u-j)=0, \end{aligned}$$

which gives

4a $$\begin{aligned} \frac{\partial w}{\partial t}&= a-w-u^2w + \nu \frac{\partial w}{\partial x}, \end{aligned}$$

4b $$\begin{aligned} \frac{\partial u}{\partial t}&= u^2w - bu + d\left( j - u\right) , \end{aligned}$$

4c $$\begin{aligned} \frac{\partial ^2 j}{\partial x^2}&= -\eta ^2(u-j). \end{aligned}$$

This ability to simplify a nonlocal dispersal term with the Laplace kernel has been exploited by many previous authors (e.g. Avitabile and Schmidt 2015; Faye 2013; Merchant and Nagata 2015; Sherratt 2016).

Bennett and Sherratt (2018) previously showed that (4) admits periodic travelling waves that represent vegetation patterns. Periodic travelling wave solutions of (4) are denoted by $W(z) = w(x,t)$, $U(z) = u(x,t)$, $J(z) = j(x,t)$ and can be expressed in terms of a single independent variable $z = x-ct$. Further denoting $M(z) = J'(z)$, periodic travelling wave solutions of (4) satisfy

5a $$\begin{aligned} (c+\nu )W'&= U^2W + W - a, \end{aligned}$$

5b $$\begin{aligned} cU'&= \alpha U - dJ - U^2W, \end{aligned}$$

5c $$\begin{aligned} J'&= M, \end{aligned}$$

5d $$\begin{aligned} M'&= \eta ^2 (J-U), \end{aligned}$$

where $\alpha = b+ d$. In the present context, transformation of the nonlocal model (2) into the local model (5) expressed in travelling wave coordinates enables calculation of the region of the *a*–*c* parameter plane in which patterns exist, and in which they are stable. Briefly, stability for a given periodic travelling wave at a fixed point in the *a*-*c* parameter plane is determined through a calculation of its essential spectrum using a numerical continuation algorithm developed by Rademacher et al. (2007). Then, by tracking properties of the essential spectrum that indicate a stability change (i.e. where it crosses the imaginary axis), stability boundaries in the *a*-*c* plane can be calculated through numerical continuation; for full details of the method see Rademacher et al. (2007), Sherratt (2012), Sherratt (2013b). This calculation was done previously by Bennett and Sherratt (2018) and their results are reproduced in Figs. 1b and 1c for convenience. Note that the numerical bifurcation work used for Fig. 1 is not currently possible for integrodifferential equations (to our knowledge), making it impossible to construct figures similar to Fig. 1b and c for other dispersal kernels.

Figure 1 shows that nonlocal seed dispersal results in the existence of spatial patterns at very low migration speeds. Moreover, for the wider dispersal case in Fig. 1c ($\eta =0.75$), the patterns at almost zero migration speeds are stable. This result was noted as an aside in Bennett and Sherratt (2018), but it was not explored in any detail. However, the result has potentially important implications, because it provides an alternative mechanism for the empirical observation of stationary (or almost stationary) vegetation patterns. The objective of this paper is to undertake a detailed investigation of these almost stationary patterns. One difficulty of the results in Fig. 1c is that they are purely numerical, and numerical continuations can sometimes indicate spurious results. Therefore, independent confirmation is essential. We will undertake an analytical study of the patterns by using a perturbation theory approach in which the migration speed *c* is taken to be a small parameter. This will provide the required independent confirmation of the existence of patterns for arbitrarily low speeds, and will also give important insight into the form of these patterns.

**Fig. 2:**
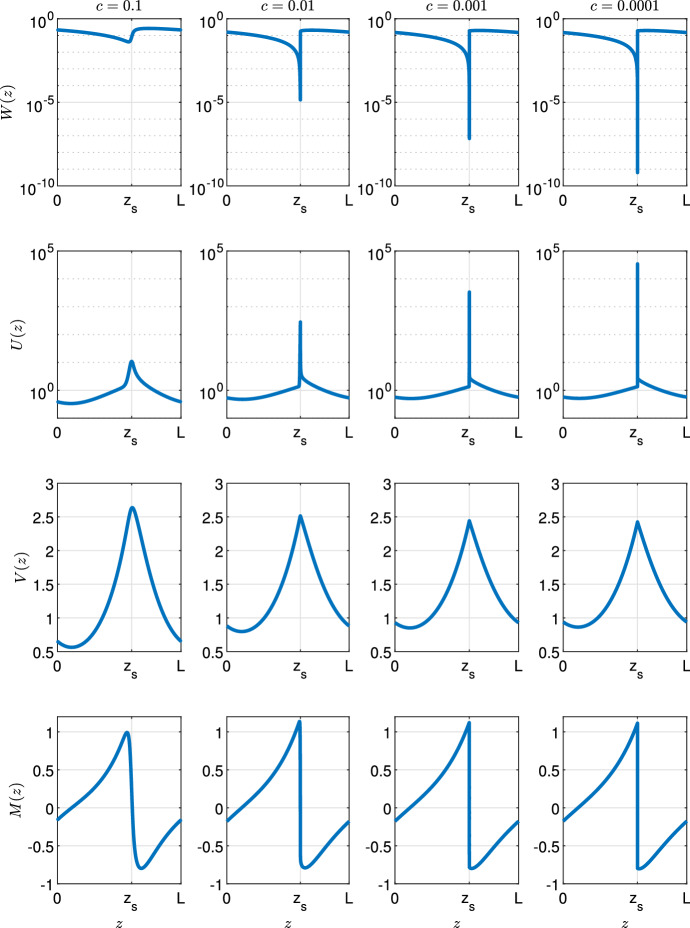
Development of spike with decreasing $$\varvec{c}$$. Solutions of (5) are shown for different values of the migration speed *c*. Solution profiles are obtained through numerical continuation and therefore do not necessarily depict stable solutions of the corresponding PDE system (2). The parameters are $$d= 0.5625, \eta = 0.75, a= 0.727, \nu = 18.25$$, and $$b= 0.45$$. Wavelength *L* is a function of the migration speed *c* and thus varies. Its value is $$L= 10.03$$, $$L= 7.85 $$, $$L= 7.37$$, and $$L= 7.26$$ for $$c = 0.1$$, $$c = 0.01$$, $$c = 0.001$$, and $$c = 0.0001$$, respectively

Before proceeding, we make a few comments on the value of the slope parameter $\nu$. In his original paper, Klausmeier (1999) estimated the value of this parameter as 182.5 for a typical dryland system. This value has been widely used in studies of the model by other authors, and it is the value used in Fig. 1. In the present paper, our focus is on small values of *c* and our analytical work applies for any value of $\nu$. However, numerical results for small *c* are more difficult to obtain and interpret when $\nu$ is large. Therefore in all subsequent numerical work we will reduce the value of $\nu$ by a factor of 10, to 18.25. An interesting future study (but well outside the scope of this paper) would be to consider a two-parameter perturbation problem in which *c* and $1/\nu$ are both small, but for our purposes $\nu$ remains fixed and finite while $c\rightarrow 0$.

**Fig. 3:**
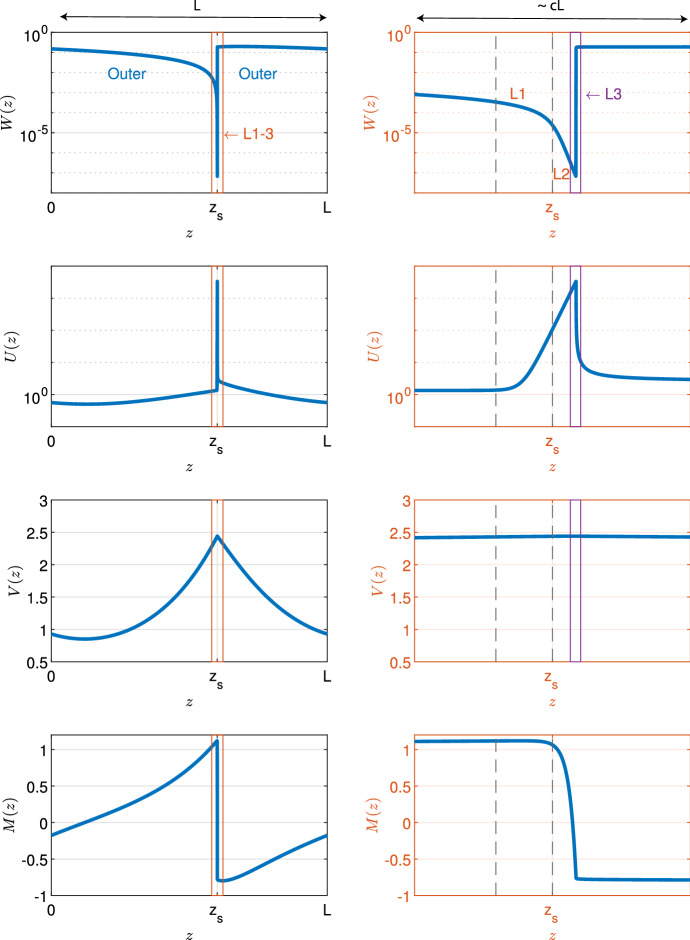
Example spike solution. An example solution of (5) obtained through numerical continuation for parameters $c = 10^{-4}, d= 0.5625, \eta = 0.75, \nu = 18.25, b= 0.45$ is shown. The first column shows the solution across a whole period for all densities; the second column shows blow-ups of the spike region. Note the logarithmic axis in plots for *W* and *U*

**Fig. 4:**
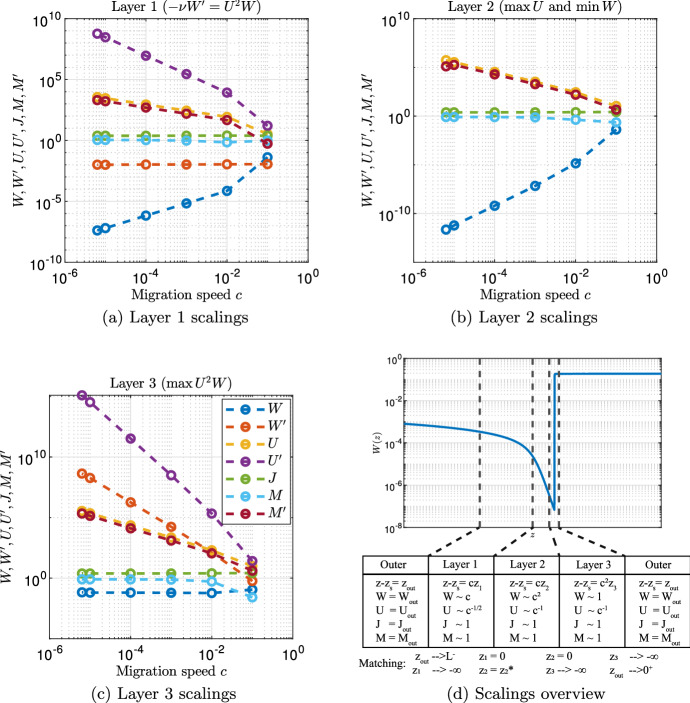
Layer scalings. The visualisations in **a**–**c** show the dependence of model densities on the migration speed *c* as observed in solutions obtained through numerical continuation. The layer solution (for *W* only) is shown in **d** alongside a sketch of the locations of the three layers. The table underneath indicates the scalings used in all layers and the *z* coordinates used for matching. Parameter values are $$d= 0.5625, \eta = 0.75, \nu = 18.25, b= 0.45$$, $$a= 0.727$$ and $$L = 7.24$$ **a**–**d** and $$c = 10^{-3} $$ (d only)

## The form of slowly moving patterns

Figure 2 illustrates the form of patterned solutions of (5) when the migration speed *c* is small. As *c* decreases with all other parameters fixed, the vegetation density *U* develops a pronounced spike (one spike per pattern wavelength), while an abrupt jump develops in the water density *W*, from almost zero to a non-zero finite value. We will show that this solution has a layered structure, with three “thin” layers, termed “layer 1”, “layer 2”, and “layer 3”, respectively, embedded in a finite-width outer solution (Fig. 3). We will develop appropriate scalings for each of the layers, and will show that matching enables the various constants of integration to be determined. Of course, the solutions of (5) depend on the values of the parameters $a$, $b$, $\nu$, $\eta$ and $d$, as well as the migration speed *c*. We will show that the solution structure we develop is only valid if $d$ is less than a critical value $d^*$. Numerical solutions for $d>d^*$ show little difference from those for smaller $d$, suggesting a (different) layer structure, and determination of this is an important target for future work.

### Layer 2

The most prominent feature of the solutions in Fig. 3 is the spike in the solution for *U*; we denote the location of this spike by $z_{s}$. As we will show below, the peak of the spike occurs in the middle layer of the three thin layers; we therefore refer to this layer as “layer 2”. Careful numerical investigation (Fig. 4b) shows that the height of this spike scales with 1/*c* as $c\rightarrow 0^+$. The scaling relevant to the increase of *U* towards the top of this spike must include the positive ($\alpha U$) term to leading order, meaning that $z-z_{s}=O_s(c)$ as $c\rightarrow 0^+$ ($f = O_s(g) \iff f = O(g)$ and $f\ne o(g)$). This implies that $U^2W\gg {}dW/dz$, and thus the only feasible leading order form for (5a) is $U^2W=a$, which requires that $W=O_s(c^2)$. This suggested rescaling is in agreement with the dependence of *W* on *c* in numerically obtained solutions (Fig. 4b). Taking these considerations together, we rewrite the model using

6a $$\begin{aligned} z - z_{s}&= cz_2, \end{aligned}$$

6b $$\begin{aligned} W(z)&=c^2W_2(z_2) + \text {h.o.t.}, \end{aligned}$$

6c $$\begin{aligned} U(z)&= c^{-1}U_2(z_2) + \text {h.o.t.}, \end{aligned}$$

6d $$\begin{aligned} J(z)&= J_2(z_2) + \text {h.o.t.}, \end{aligned}$$

6e $$\begin{aligned} M(z)&= M_2(z_2) + \text {h.o.t.}. \end{aligned}$$

Here, and throughout the paper, we use “$\text {h.o.t.}$” to denote “higher order terms”. Substitution of (6) into (5) gives $U_2^2W_2 = a$, $U_2' = \alpha U_2$, $J_2' = 0$, and $M_2' = -\eta ^2 U_2$ to leading order as $c \rightarrow 0^+$. Therefore, the leading order solution in layer 2 is

7a $$\begin{aligned} W_2(z_2)&= \frac{a}{k_1^2}e^{-2\alpha z_2}, \end{aligned}$$

7b $$\begin{aligned} U_2(z_2)&= k_1e^{\alpha z_2}, \end{aligned}$$

7c $$\begin{aligned} J_2(z_2)&\equiv k_2, \end{aligned}$$

7d $$\begin{aligned} M_2(z_2)&= k_3- \frac{\eta ^2 k_1}{\alpha }e^{\alpha z_2}. \end{aligned}$$

Here and throughout the manuscript, $k_i \in \mathbb {R}, i \in \mathbb {N}$ denote constants of integration that will be determined by matching the solutions in the various layers (Sect. 3.5).

### Layer 3

To the right of layer 2, *U* decreases in a separate layer, here termed “layer 3”. The above rescalings for layer 2 give leading order equations that imply $U'>0$. Therefore, the decrease in *U* from its maximum at the top of the spike must be governed by a different set of scalings, which must nevertheless have $U=O_s(1/c)$. The $-U^2W$ term must be present to leading order (in order for *U* to decrease), so that $W\cdot (z-z_{s})=O_s(c^2)$. To avoid the “$-a$” term dominating in (5a), we must have $W' = O_s(U^2W) \Rightarrow z-z_{s}=O(c^2)$ and $W=O_s(1)$. These considerations are in agreement with the dependence of model densities on *c* in layer 3 (Fig. 4c) Hence we rewrite the model densities as

8a $$\begin{aligned} z - z_{s}&= c^2z_3, \end{aligned}$$

8b $$\begin{aligned} W(z)&= W_3^0(z_3) + cW_3^1 + \text {h.o.t.}, \end{aligned}$$

8c $$\begin{aligned} U(z)&= c^{-1}U_3^{-1}(z_3) + U_3^0 + \text {h.o.t.}, \end{aligned}$$

8d $$\begin{aligned} J(z)&= J_3(z_3) + \text {h.o.t.}, \end{aligned}$$

8e $$\begin{aligned} M(z)&= M_3(z_3) + \text {h.o.t.}. \end{aligned}$$

Substitution of (8) into (5) gives $\nu (W_3^{0})' = (U_3^{-1})^2W_3^0$, $(U_3^{-1})' = -(U_3^{-1})^2W_3^0$, $J_3' = 0$, and $M_3' = 0$ to leading order as $c \rightarrow 0^+$. Therefore, $J_3(z_3) \equiv k_4$ and $M_3(z_3) \equiv k_5$. The solution of the remaining two equations can only be obtained implicitly. We have $\nu (W_3^{0})' + (U_3^{-1})' = 0$ and therefore ${\nu }W_3^{0} + U_3^{-1} = k_6$, say. Hence, $\nu (U_3^{-1})' = -(U_3^{-1})^2 (k_6-U_3^{-1})$, which can be solved to give

9 $$\begin{aligned} k_7+ \frac{1}{\nu }z_3 = \int \frac{1}{(U_3^{-1})^2\left( U_3^{-1} - k_6\right) } = \frac{1}{k_6U_3^{-1}} - \frac{1}{k_6^2}\log \left( \frac{U_3^{-1}}{k_6- U_3^{-1}}\right) . \end{aligned}$$

As we show below (see Sect. 3.5.4), the layer 3 leading order solutions for *W* and *U* are insufficient for matching to the outer solution. Therefore, we also require the first order correction terms; this is in contrast to both layer 2 (discussed above) and layer 1 (discussed below) where leading order behaviour suffices. Substituting (8) into equations for *W* and *U* in (5) and retaining first order correction terms yields

$$\begin{aligned} (U_3^0)'&= \alpha U_3^{-1} - (U_3^{-1})^2W_3^1 - 2U_3^{-1}U_3^0W_3^0, \\ (W_3^0)' + \nu (W_3^1)'&= (U_3^{-1})^2W_3^1 + 2U_3^{-1}U_3^0W_3^0, \end{aligned}$$

where we have used $(U_3^{-1})' = -(U_3^{-1})^2W_3^0$, $\nu (W_3^0)' = (U_3^{-1})^2W_3^0$, and $U_3^{-1} + \nu W_3^0 = k_6$. Division by $(U_3^{-1})' = -(U_3^{-1})^2W_3^0$ gives

10a $$\begin{aligned} \frac{{{\text {d}}}U_3^0}{{{\text {d}}}U_3^{-1}}&= -\frac{\alpha }{U_3^{-1}W_3^0} + \frac{W_3^1}{W_3^0} + 2\frac{U_3^0}{U_3^{-1}}, \end{aligned}$$

10b $$\begin{aligned} -\frac{1}{\nu } + \nu \frac{{{\text {d}}}W_3^1}{{{\text {d}}}U_3^{-1}}&= - \frac{W_3^1}{W_3^0} - 2\frac{U_3^0}{U_3^{-1}}. \end{aligned}$$

Addition of both equations yields

$$\begin{aligned} \frac{{{\text {d}}}}{{{\text {d}}}U_3^{-1} } \left( U_3^ 0 + \nu W_3^1\right) = \frac{1}{\nu } - \frac{\alpha }{U_3^{-1}W_3^0} = \frac{1}{\nu } - \frac{\alpha \nu }{U_3^{-1}\left( k_6- U_3^{-1}\right) }, \end{aligned}$$

and therefore

$$\begin{aligned} U_3^ 0 + \nu W_3^1 =k_8+ \frac{U_3^{-1}}{\nu } - \frac{\alpha \nu }{k_6}\log \left( \frac{U_3^{-1}}{k_6- U_3^{-1}}\right) . \end{aligned}$$

Thus, substitution into (10a) gives

$$\begin{aligned} \frac{{{\text {d}}}U_3^0}{{{\text {d}}}U_3^{-1}} - 2\frac{U_3^0}{U_3^{-1}} + \frac{U_3^0}{k_6- U_3^{-1}}&= - \frac{\alpha \nu }{U_3^{-1}\left( k_6- U_3^{-1}\right) } + \frac{k_8}{k_6- U_3^{-1}} \\&\quad + \frac{U_3^{-1}}{\nu \left( k_6- U_3^{-1} \right) } - \frac{\alpha \nu \log \left( \frac{U_3^{-1}}{k_6- U_3^{-1}}\right) }{k_6\left( k_6- U_3^{-1}\right) }, \end{aligned}$$

and, after algebraic manipulation,

11 $$\begin{aligned} \frac{{{\text {d}}}}{{{\text {d}}}U_3^{-1}} \left( \frac{U_3^0}{(U_3^{-1})^2}\right)= & {} - \frac{\alpha \nu }{(U_3^{-1})^3\left( k_6- U_3^{-1}\right) } + \frac{k_8}{(U_3^{-1})^2\left( k_6- U_3^{-1}\right) }\nonumber \\{} & {} + \frac{1}{\nu U_3^{-1}\left( k_6- U_3^{-1}\right) } - \frac{\alpha \nu \log \left( \frac{U_3^{-1}}{k_6- U_3^{-1}}\right) }{k_6(U_3^{-1})^2\left( k_6- U_3^{-1}\right) }. \end{aligned}$$

### Layer 1

In layer 2, *W* increases towards $\infty$ exponentially as $z_2\rightarrow -\infty$, which prevents direct matching with the outer solution. Therefore, an intermediate layer, here termed “layer 1”, is required, in which $1\ll U \ll (1/c)$ and $1\gg W \gg c^2$. The only possible scaling is $U=O(c^{-1/2})$ and $W=O(c)$, which in turn requires $z-z_{s}=O_s(c)$. Similar to the other layers, these considerations are corroborated by a numerical investigation of model solutions (Fig. 4a). Hence we rewrite the model densities as

12a $$\begin{aligned} z - z_{s}&= cz_1, \end{aligned}$$

12b $$\begin{aligned} W(z)&=cW_1(z_1) + \text {h.o.t.}, \end{aligned}$$

12c $$\begin{aligned} U(z)&= c^{-\frac{1}{2}}U_1(z_1) + \text {h.o.t.}, \end{aligned}$$

12d $$\begin{aligned} J(z)&= J_1(z_1) + \text {h.o.t.}, \end{aligned}$$

12e $$\begin{aligned} M(z)&= M_1(z_1) + \text {h.o.t.}. \end{aligned}$$

Substitution of (12) into (5) gives $\nu W_1' = U_1^2W_1-a$, $U_1' = \alpha U_1$, $J_1' = 0$, and $M_1' = 0$ to leading order as $c \rightarrow 0^+$. Therefore, the leading order solution in layer 1 is

13a $$\begin{aligned} W_1(z_1)&= e^\xi \left( k_9- \frac{a}{2\nu \alpha } E(\xi ) \right) , \end{aligned}$$

13b $$\begin{aligned} U_1(z_1)&= k_{10}e^{\alpha z_1}, \end{aligned}$$

13c $$\begin{aligned} J_1(z_1)&\equiv k_{11}, \end{aligned}$$

13d $$\begin{aligned} M_1(z_1)&\equiv k_{2}, \end{aligned}$$

where

13e $$\begin{aligned} \xi = \frac{k_{10}^2}{2\nu \alpha } e^{2\alpha z_1}, \quad \text {and} \quad E(\xi ) = \int \frac{e^{-\xi }}{\xi } {{\text {d}}}\xi , \end{aligned}$$

denotes the exponential integral.

Note that the scalings for *z* are the same in the adjacent layers 1 and 2. This implies an abrupt transition between the two layers at finite values of $z_1$ and $z_2$.

### Outer solution

Away from the spike, the dynamics for $c \ll 1$ are identical to those of the system with $c=0$, the so called “outer system”. To analyse the outer system, we write $z-z_{s}= z_{{{\text {out}}}}$, $U(z) = U_{{\text {out}}}(z_{{\text {out}}})$, $W(z)=W_{{\text {out}}}(z_{{\text {out}}})$, $J(z) = J_{{\text {out}}}(z_{{\text {out}}})$ and $M(z) = M_{{\text {out}}}(z_{{\text {out}}})$ so that in the new variables, the layers are located at the boundary of the outer domain. Substituting $c=0$ into (5) then yields

14a $$\begin{aligned} \nu W_{{\text {out}}}'&= U_{{\text {out}}}^2W_{{\text {out}}}+ W_{{\text {out}}}- a, \end{aligned}$$

14b $$\begin{aligned} 0&= \alpha U_{{\text {out}}}- dJ_{{\text {out}}}- U_{{\text {out}}}^2W_{{\text {out}}}, \end{aligned}$$

14c $$\begin{aligned} J_{{\text {out}}}'&= M_{{\text {out}}}, \end{aligned}$$

14d $$\begin{aligned} M_{{\text {out}}}'&= \eta ^2 (J_{{\text {out}}}-U_{{\text {out}}}). \end{aligned}$$

Note that $c=0$ reduces the system of equations to a third order ODE system given by (14a), (14c) and (14d), which is equipped with the algebraic relation (14b). The algebraic relation is a quadratic in $U_{{\text {out}}}$, which can be solved to give

$$\begin{aligned} U_{{\text {out}}}^\pm = \frac{1}{2W_{{\text {out}}}} \left( \alpha \pm \sqrt{\alpha ^2 - 4dJ_{{\text {out}}}W_{{\text {out}}}} \right) . \end{aligned}$$

**Fig. 5:**
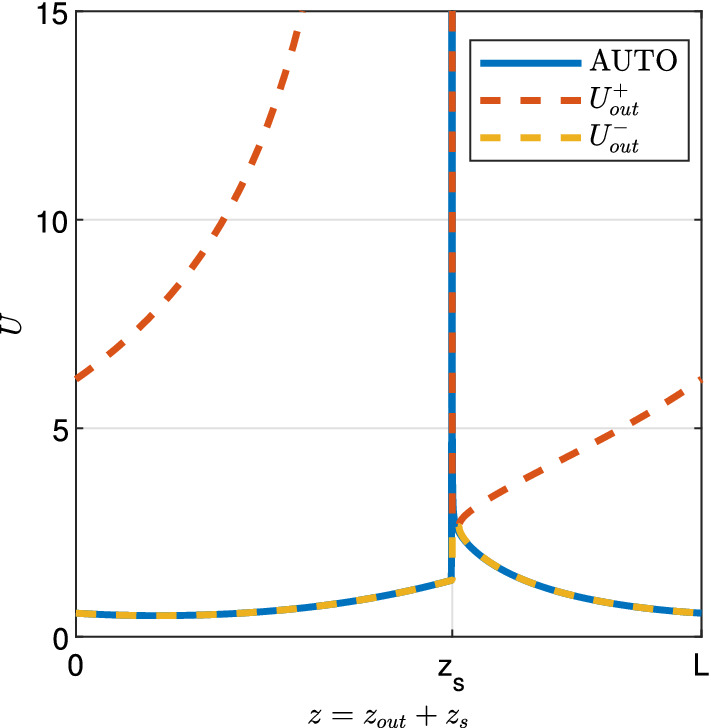
Outer solution for $\varvec{U}$. A visualisation of the solution for *U* obtained through numerical continuation is compared with $U_{{\text {out}}}^+$ and $U_{{\text {out}}}^-$, the two solutions of the algebraic equation (14b). Note that only $U_{{\text {out}}}^-$ shows good agreement with the AUTO solution away from the spike. Parameter values are $c = 10^{-3}, d= 0.5625, \eta = 0.75, \nu = 18.25, b= 0.45$, $a= 0.727$ and $L = 7.24$

A comparison of $U_{{\text {out}}}^+$ and $U_{{\text {out}}}^-$ with solutions obtained through numerical continuation shows that $U_{{\text {out}}}= U_{{\text {out}}}^-$ throughout the entire outer solution (Fig. 5). We thus use $U_{{\text {out}}}= U_{{\text {out}}}^-$ in (14). The resulting system is a third order ODE system for $W_{{\text {out}}}(z_{{\text {out}}})$, $J_{{\text {out}}}(z_{{\text {out}}})$ and $M_{{\text {out}}}(z_{{\text {out}}})$ for $z_{{\text {out}}}\in [0,L]$. To analyse the system, we treat it as a boundary value problem that can be solved numerically (Sect. 3.6). Suitable boundary conditions are determined through matching the outer solution to the layer solutions (see Section 3.5) and are given by (21a).

### Matching

The layer solutions obtained in the preceding sections contain constants of integration. These constants can be obtained by matching the layer solutions with each other and the outer solution.

#### Matching between outer solution and layer 1

Matching between the outer layer and layer 1 occurs as $z_{{\text {out}}}\rightarrow L^-$ and $z_1 \rightarrow -\infty$, respectively. For brevity, we write $\lim _{z_{{\text {out}}}\rightarrow L^-}W_{{\text {out}}}(z_{{\text {out}}}) = W_L$, $\lim _{z_{{\text {out}}}\rightarrow L^-}U_{{\text {out}}}(z_{{\text {out}}}) = U_L$, $\lim _{z_{{\text {out}}}\rightarrow L^-}J_{{\text {out}}}(z_{{\text {out}}}) = J_L$, and $\lim _{z_{{\text {out}}}\rightarrow L^-}M_{{\text {out}}}(z_{{\text {out}}}) = M_L$. From (13), we have $\xi \rightarrow 0^+$ as $z_1 \rightarrow -\infty$ and therefore $E(\xi ) = -\log (\xi ) - \gamma + O(\xi ) = -2\alpha z_1 + O(1)$, where $\gamma$ denotes the Euler–Mascheroni constant. This yieldsThus, we have $W(z) = cW_1(z_1) = -\frac{a}{\nu } (z-z_{s})$ to leading order in *c* as $c \rightarrow 0^+$. Therefore, leading order matching requires $W_L = 0$ in the outer solution. Leading order matching for *U* is automatic because $U \sim c^{-1/2}$ in layer 1 and $U_1 \rightarrow 0$ as $z_1 \rightarrow -\infty$. Finally, leading order matching requires $J_L = k_{11}$ and $M_L = k_{2}$.

#### Matching between layer 1 and layer 2

Matching between layer 1 and layer 2 occurs at $z_1 = 0$ and $z_2 = z_2^*(c)$, respectively.

Due to the different scalings in both *W* and *U* between the layers, matching requires that either (i) $U_1$ and $W_2$ become infinite at the matching values or (ii) the matching locus depends on the migration speed *c*. Our calculations in Sect. 3.3 and 3.1 show that $U_1$ and $W_2$ remain finite for finite $z_1$ and $z_2$, respectively. Therefore, matching requires dependence of one of the matching loci on *c*; here the appropriate relation is that $z_2^*(c) \sim \log (c)$.

For layer 1, the matching location is arbitrary and we choose $z_1 = 0$ for mathematical convenience. From (13), we have

15 $$\begin{aligned} {\begin{matrix} W_1(0) &{}= e^{\frac{k_{10}^2}{2\alpha \nu }} \left( k_9+ \frac{a}{2 \alpha \nu } E\left( \frac{k_{10}^2}{2\alpha \nu } \right) \right) , \\ U_1(0) &{}= k_{10}, \\ J_1(0) &{}= k_{11}, \\ M_1(0) &{}= k_{2}. \end{matrix}} \end{aligned}$$

As outlined above, the appropriate choice for the matching location in layer 2 is $z_2^* = \frac{1}{2\alpha }\log c + \beta$, $\beta \in \mathbb {R}$, noting the dependence on the migration speed *c*. From (7), we have

16 $$\begin{aligned} {\begin{matrix} W_2\left( z_2^*\right) &{}= c^{-1} \frac{a}{k_1^2}e^{-2\alpha \beta }, \\ U_2\left( z_2^*\right) &{}= c^{\frac{1}{2}}k_1e^{\alpha \beta }, \\ J_2\left( z_2^*\right) &{}= k_2, \\ M_2\left( z_2^*\right) &{}= k_3- c^{\frac{1}{2}}\frac{\eta ^2 k_1}{\alpha }e^{\alpha \beta }. \end{matrix}} \end{aligned}$$

Therefore, recalling that $W \sim c$ in layer 1 and $W \sim c^2$ in layer 2, leading order matching for *W* requires $cW_1(0) = c^2W_2(z_2^*)$ which yields

$$\begin{aligned} k_9= \frac{a}{k_1^2}e^{-2\alpha \beta - \frac{k_{10}^2}{2\alpha \nu }} - \frac{a}{2\alpha \nu } E\left( \frac{k_{10}^2}{2\alpha \nu }\right) . \end{aligned}$$

Similarly, recalling that $U \sim c^{-1/2}$ in layer 1 and $U \sim c^{-1}$ in layer 2, leading order matching for *U* requires $c^{-1/2}U_1(0) = c^{-1}U_2(z_2^*)$, which yields $k_{10}= k_1e^{\alpha \beta }$. Moreover, leading order matching for *J* requires $k_{11}= k_2$ and leading order matching for *M* requires $k_{2}= k_3$, noting that $M_2(z_2^*) \rightarrow k_3$ as $c \rightarrow 0^+$.

#### Matching between layer 2 and layer 3

Matching between layer 2 and layer 3 occurs at a finite value of $z_2$, here chosen to be $z_2 = 0$, and $z_3 \rightarrow -\infty$ due to the differences in *z* scalings across both layers. From (7), we have

17 $$\begin{aligned} {\begin{matrix} W_2\left( 0\right) &{}= \frac{a}{k_1^2}, \\ U_2\left( 0\right) &{}= k_1, \\ J_2\left( 0\right) &{}= k_2, \\ M_2\left( 0\right) &{}= k_3- \frac{\eta ^2 k_1}{\alpha }, \end{matrix}} \end{aligned}$$

for the layer 2 solutions. Similarly, from (9), we have18in layer 3.

Hence, leading order matching for *W* is automatic because $W \sim c^2$ in layer 2, $W \sim 1$ in layer 3 and $W_3^0 \rightarrow 0$ as $z_3 \rightarrow -\infty$. For all other variables, the scalings in layers 2 and 3 are identical. Thus, leading order matching requires $U_2(0) = \lim _{z_3\rightarrow -\infty } U_3^{-1}(z_3)$ and similarly for *J* and *M*. These lead to the conditions $k_1= k_6$, $k_2= k_4$ and $k_5= k_3- \frac{\eta ^2 k_1}{\alpha }$.

#### Matching between layer 3 and outer solution

Finally, matching between layer 3 and the outer solution occurs at $z_3 \rightarrow \infty$ and $z_{{\text {out}}}\rightarrow 0^+$, respectively. For brevity, we write $\lim _{z_{{\text {out}}}\rightarrow 0^+}W_{{\text {out}}}(z_{{\text {out}}}) = W_0$, $\lim _{z_{{\text {out}}}\rightarrow 0^+}U_{{\text {out}}}(z_{{\text {out}}}) = U_0$, $\lim _{z_{{\text {out}}}\rightarrow 0^+}J_{{\text {out}}}(z_{{\text {out}}}) = J_0$, and $\lim _{z_{{\text {out}}}\rightarrow 0^+}M_{{\text {out}}}(z_{{\text {out}}}) = M_0$. In layer 3, from (9), we have19Since $W\sim 1$ in layer 3 and $W_3^0 \rightarrow k_6/\nu$ as $z_3 \rightarrow \infty$, we require $W_0 = k_6/\nu$. Due to a similar argument, leading order matching also requires $J_0 = k_4$. Combined with conditions derived above, this yields $J_0 = J_L$. Similarly, we require $M_0 = k_5$ which yields $M_L = M_0 - \frac{\eta ^2W_0}{\alpha }$ after combining with conditions derived from matching other layers. For *U*, leading order matching would require $U_0 = 0$, because

$$\begin{aligned} U(z) = c^{-1}U_3^{-1}(z_3) \sim \frac{\nu }{ck_6z_3} = \frac{c\nu }{k_6z} \rightarrow 0 \quad \text {as} \quad c \rightarrow 0^+. \end{aligned}$$

However, from the algebraic equation (14b) in the outer system, we have

$$\begin{aligned} U_0 = U_{{\text {out}}}(0)&= U_{{\text {out}}}^-(0) = \frac{1}{W_{{\text {out}}}(0)}\left( \alpha \pm \sqrt{\alpha ^2 - 4dJ_{{\text {out}}}(0) W_{{\text {out}}}(0)} \right) \\&= \frac{\nu }{2k_6}\left( \alpha \pm \sqrt{\alpha ^2 - \frac{4dk_6}{\nu }} \right) \ne 0. \end{aligned}$$

Thus, we need to consider the first order correction, $U_3^0$. Recall that $U_3^{-1} \rightarrow 0$ as $z_3 \rightarrow \infty$. Hence, the right hand side of (11) behaves like $\frac{\alpha \nu }{k_6(U_3^{-1})^3}$ to leading order as $z_3 \rightarrow \infty$. Therefore, we obtain the leading order equation

$$\begin{aligned} \frac{U_3^0}{(U_3^{-1})^2} = \frac{\alpha \nu }{k_6(U_3^{-1})^2}. \end{aligned}$$

From this, we obtain that

20 $$\begin{aligned} U_3^0 \rightarrow \frac{\alpha \nu }{2k_6}, \quad \text {as} \quad z_3 \rightarrow \infty . \end{aligned}$$

Combined with the leading order term, this yields

$$\begin{aligned} U(z) = c^{-1}U_3^{-1}(z_3) + U_3^0(z_3) \rightarrow \frac{\alpha \nu }{2k_6} \quad \text {as} \quad c \rightarrow 0^+. \end{aligned}$$

Therefore, matching requires $U_0 = \frac{\alpha \nu }{2k_6}$. Combined with (14b), this yields $W_0J_0 = \frac{\alpha ^2}{4d}$.

#### Matching summary

Combined, the considerations of this section lead to the following matching conditions:

21a $$\begin{aligned} W_L&= 0, \quad W_0J_0 = \frac{\alpha ^2}{4d}, \quad J_L = J_0, \quad M_L = M_0 - \frac{\eta ^2W_0}{\alpha }, \end{aligned}$$

21b $$\begin{aligned} k_1= k_6&= \nu W_0, \quad k_{10}= k_6e^{\alpha \beta }, \quad k_9= \frac{a}{k_1^2}e^{-2\alpha \beta - \frac{k_{10}^2}{2\alpha \nu }} - \frac{a}{2\alpha \nu } E\left( \frac{k_{10}^2}{2\alpha \nu }\right) , \end{aligned}$$

21c $$\begin{aligned} k_{11}&= k_2= k_4= J_0, \quad k_{2}= k_3= k_5- \frac{\eta ^2k_1}{\alpha } = M_L. \end{aligned}$$

Note that (21a) determine the boundary conditions for the outer system (14) in terms of model parameters (i.e., no information about the layered solution is required). Conditions (21b) and (21c) fully determine all constants of integration in all three layers, noting that information on the outer solution is required.

### Solution of outer equations

The outer equations (14) are a system of three coupled ODEs, with the complication that $U_{{\text {out}}}$ depends on the equation variables via a quadratic polynomial. We have been unable to solve these equations analytically, but numerical solution of the boundary value problem (14,21a) is straightforward. We used a solution from our numerical continuation as an initial guess for one reference set of parameters, and then transitioned to the required parameters via bootstrapping. This procedure revealed an important and unexpected result: solution is only possible if *d* is sufficiently small.

**Fig. 6:**
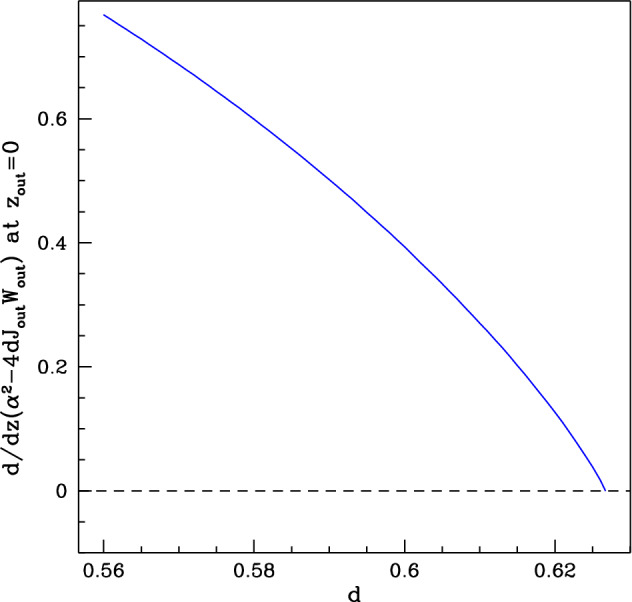
Derivative of the discriminant of the quadratic for $\varvec{U}_{{\text {out}}}$. The derivative of $\alpha ^2 - 4dJ_{{\text {out}}}W_{{\text {out}}}$ evaluated at $z_{{\text {out}}}= 0$ is plotted against $d$. Note that the derivative becomes zero at at $d= d^*$. The densities $w_{{\text {out}}}$ and $J_{{\text {out}}}$ were obtained by solving the boundary value problem (14,21a) with parameter values $\eta = 0.75, \nu = 18.25, b= 0.45$, $a= 0.727$ and $L = 7.24$

The explanation for this lies in the form of the solution at $z_{{\text {out}}}=0$. One of the boundary conditions specifies $W_{{\text {out}}}J_{{\text {out}}}=\alpha ^2/4d$ at this boundary. Also (14b) imposes the requirement $W_{{\text {out}}}J_{{\text {out}}}\ge \alpha ^2/4d$ for all $z_{{\text {out}}}$. Therefore, a necessary condition for the boundary value problem to have a (real-valued) solution is ${{\text {d}}}/{{\text {d}}}z(W_{{\text {out}}}J_{{\text {out}}})\ge 0$ at $z_{{\text {out}}}=0$ to guarantee $U_{{\text {out}}}\in \mathbb {R}$. Figure 6 shows a plot of ${{\text {d}}}/{{\text {d}}}z (\alpha ^2 - 4dW_{{\text {out}}}J_{{\text {out}}})$ at $z_{{\text {out}}}=0$ (calculated numerically) as a function of $d$, for fixed values of the other parameters. The derivative decreases as $d$ increases, reaching zero at a finite value $d=d^*$; there does not appear to be a real-valued solution of (14,21a). It is important to emphasise that these conclusions are based on numerical calculations rather than analysis, but they suggest that a different solution structure applies for $d>d^*$.

### Construction of composite solution

With the leading order behaviour of solutions in the layers derived and matching conditions between layers and to the outer solution obtained, we can now construct a composite solution. For this, it is convenient to represent the solution in vector form, i.e. we write $\textbf{Y}(z) = [W(z), U(z), J(z), M(z)]^T$. Similarly, we also represent the outer solution and layer solutions in vector form and write $\textbf{Y}_{{\text {out}}}(z_{{\text {out}}}) = [W_{{\text {out}}}(z_{{\text {out}}}), U_{{\text {out}}}(z_{{\text {out}}}), J_{{\text {out}}}(z_{{\text {out}}}), M_{{\text {out}}}(z_{{\text {out}}})]^T$, $\textbf{Y}_1(z_1) = [cW_1(z_1), c^{-1/2}U_1(z_1), J_1(z_1), M_1(z_1)]^T$, $\textbf{Y}_2(z_2) = [c^2W_2(z_2), c^{-1}U_2(z_2), J_2(z_2),M_2(z_2)]^T$, and $\textbf{Y}_3(z_3) = [W_3(z_3), c^{-1}U_3(z_3), J_3(z_3), M_3(z_3)]^T$. Finally, we also define vectors containing information on the boundary of the outer solution, i.e. we set $\textbf{Y}_L = \lim _{z_{{\text {out}}}\rightarrow L^-}\textbf{Y}_{{\text {out}}}(z_{{\text {out}}}) = [W_L,U_L,J_L,M_L]^T$ and $\textbf{Y}_0 = \lim _{z_{{\text {out}}}\rightarrow 0^+}\textbf{Y}_{{\text {out}}}(z_{{\text {out}}}) = [W_0,U_0,J_0,M_0]^T$.

Using this notation, we define the composite solution as

$$\begin{aligned} \textbf{Y}(z) = {\left\{ \begin{array}{ll} \textbf{Y}_{{\text {out}}}(\tilde{z}+L-z_{s}) + \textbf{Y}_1\left( \dfrac{\tilde{z}-z_{s}}{c}\right) + \textbf{Y}_3\left( \dfrac{\tilde{z}+L-z_{s}}{c^2}\right) - \textbf{Y}_L - \textbf{Y}_0 &{}\text { if } \dfrac{c\log {c}}{2\alpha } \le z \le z_{s}+ \dfrac{c\log {c}}{2\alpha } \\ \textbf{Y}_2(z_2) &{}\text { if } z_{s}+ \dfrac{c\log {c}}{2\alpha } \le z \le z_{s}\\ \textbf{Y}_{{\text {out}}}(z-z_{s}) + \textbf{Y}_1\left( \dfrac{z-L-z_{s}}{c}\right) + \textbf{Y}_3\left( \dfrac{z-z_{s}}{c^2}\right) - \textbf{Y}_L - \textbf{Y}_0 &{}\text { if } z_{s}\le z \le L \end{array}\right. }, \end{aligned}$$

where $$\tilde{z} = z - \frac{c\log {c}}{2\alpha }$$.

**Fig. 7:**
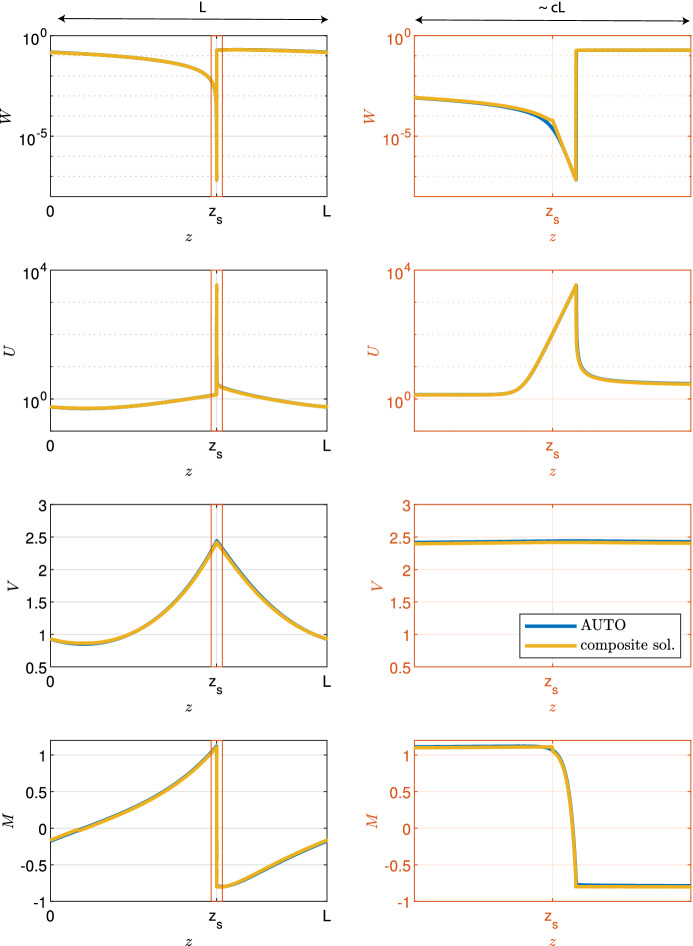
Comparison of composite solution with solution obtained through numerical continuation. An example solution of (5) obtained through numerical continuation (blue) is compared to the constructed composite solution (yellow). The parameters are $c = 10^{-3}, d= 0.5625, \eta = 0.75, \nu = 18.25, b= 0.45$, $a= 0.727$ and $L = 7.24$. The first column shows the solution across a whole period for all densities; the second column shows blow-ups of the spike region. Note the logarithmic axis in plots for *W* and *U* (color figure online)

This construction splits the composite solution into two distinct zones. First, in the region in which $\frac{c\log {c}}{2\alpha } \le z \le z_{s}+ \frac{c\log {c}}{2\alpha }$ or $z_{s}\le z \le L$ (note that this forms one connected region because $\textbf{Y}$ is the approximation of one period of a periodic travelling wave solution), the composite solution is given by the outer solution $\textbf{Y}_{{\text {out}}}(z_{{\text {out}}})$, the layer 1 solution $\textbf{Y}_1(z_1)$ and layer 3 solution $\textbf{Y}_3(z_3)$, with appropriate duplicate terms ($\textbf{Y}_L$ and $\textbf{Y}_0$), obtained through the matching process, subtracted. Second, the layer 2 solution $\textbf{Y}_2(z_2)$ acts as a bridge to cover the gap $z_{s}+ \frac{c\log {c}}{2\alpha } \le z \le z_{s}$. Note that this construction results in a composite solution with period $L - \frac{c\log {c}}{2\alpha }$ that is larger than the true period *L*. However, the error in the period is $O(c|\log {c}|)$ and thus tends to zero as $c \rightarrow 0^+$. We observe a good fit between the composite solution and the solution obtained through numerical continuation (Fig. 7).

## Solution properties

**Fig. 8:**
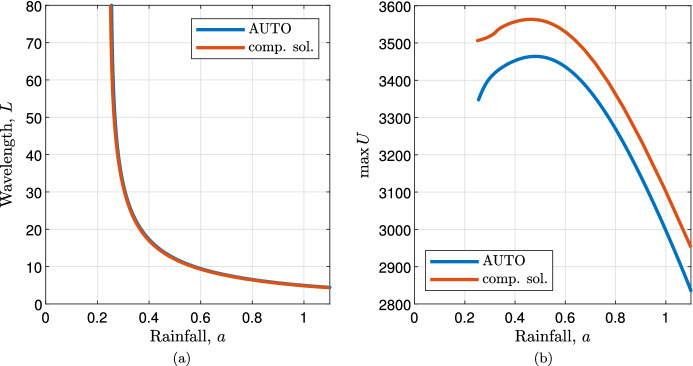
Solution properties. Wavelength (**a**) and the height of the spike (**b**) obtained from the constructed composite solution are compared with data obtained through numerical continuation. The parameter values are $c = 10^{-3}, d= 0.5625, \eta = 0.75, \nu = 18.25, b= 0.45$

Having constructed a composite solution analytically, we are able to analyse key solution properties without the need to rely on numerical simulations or numerical continuation of the full system (2). Indeed, comparison of data obtained from the composite solution is in good agreement with data obtained using numerical continuation (Fig. 8).

Numerically solving the boundary value problem (“outer system”) (14) equipped with (21a) provides information on the wavelength of spike solutions of (2). Similar to results for the local model (1), the solution wavelength decreases *L* with increasing rainfall $a$ (Fig. 8a).

The relation between the height of the spike $\max U$ and rainfall $a$ is not monotonic. The height of the spike attains its maximum for intermediate rainfall levels and decreases on either side of this maximum within the existence region of the spike solution (Fig. 8b).

## Discussion

Uphill migration of banded vegetation patterns is predicted by a variety of mathematical models in which diffusion is used to represent plant dispersal. However, diffusion fails to reflect the long-range nature of dispersal in the majority of plant species, and a dispersal kernel in an integral term is more realistic (Nathan et al. 2012; Bullock et al. 2017). Our key result in this paper is that such long-range dispersal can lead to almost stationary banded vegetation patterns. This is consistent with remote-sensing studies, which provide clear evidence of migration in some instances of banded vegetation, but find no detectable movement in other cases (Deblauwe et al. 2012; Tongway and Ludwig 2001).

**Fig. 9:**
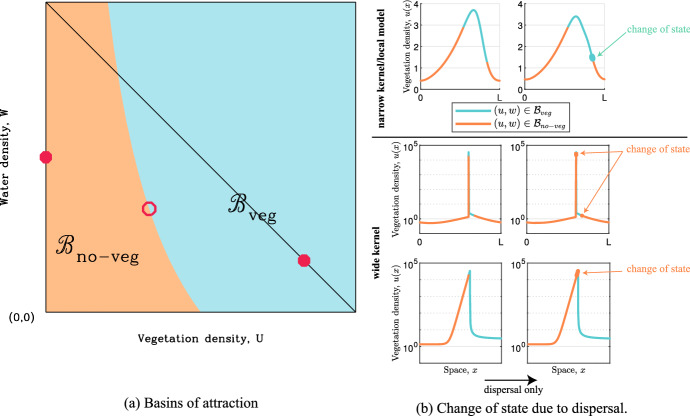
Basins of attraction explain occurrence of almost stationary patterns for wide dispersal kernels. Basins of attraction in the absence of spatial dynamics are shown schematically in (**a**). Closed circles indicate stable steady states. The open circle indicates an unstable steady state which is a saddle point. The basins of attraction are separated by the two solution trajectories that terminate at the saddle point. The left column in (**b**) shows the plant densities of two solutions classified into which basin of attraction it belongs to for the local model (1) (top) and nonlocal model (2) (bottom) with wide dispersal kernel. For the nonlocal model, both the whole spatial domain and a blow-up of the spike region are shown. Note that the classification also depends on the water density (not shown). The column on the right in (**b**) shows changes to the plant density due to dispersal over a short time interval in the absence of any non-spatial dynamics. Bold lines indicate regions in which the classification changes due to dispersal. Note the logarithmic axis and restriction of the field of view to the spike region in the bottom row

There is a clear biological basis for the uphill migration of vegetation patterns. The downhill flow of rainwater runoff causes the upslope edge of a vegetation band to be wetter than the downslope edge, causing lower plant mortality and higher seed germination rates (Tongway and Ludwig 2001; Valentin et al. 1999). This results in uphill migration on the timescale of the plant generation time. Therefore, our results demand an intuitive/biological explanation for why migration can be negligible or absent for long-range plant dispersal. The key to this explanation lies in the temporal dynamics of the model (2). In the absence of spatial variation, the equations comprise a system of two coupled ordinary differential equations, whose phase plane is illustrated schematically in Fig. 9a. There are two stable steady states, one vegetated and the other without vegetation. We denote the basins of attraction of these two steady states by $\mathcal{B}_{\textrm{veg}}$ and $\mathcal{B}_{\mathrm{no-veg}}$ respectively; they are delineated by a separatrix that passes through the third (unstable) steady state. Although these basins of attraction apply strictly only in the absence of spatial variation, they also provide a useful intuitive basis for understanding pattern migration.

Consider first a banded vegetation pattern with local (diffusive) plant dispersal (Fig. 9b, top). In the peaks of the vegetation pattern the values of plant and water density are within $\mathcal{B}_{\textrm{veg}}$, while in the troughs they are in $\mathcal{B}_{\mathrm{no-veg}}$. In the absence of plant growth and water addition/removal dynamics, (local) plant dispersal increases the plant density at the periphery of the vegetation band. The (relatively) high water level on the uphill side means that this increased plant density leads to a transition of the solution from $\mathcal{B}_{\mathrm{no-veg}}$to $\mathcal{B}_{\textrm{veg}}$, while the lower water level on the downhill side means the solution remains in $\mathcal{B}_{\mathrm{no-veg}}$(Fig. 9b, top). The result in an uphill migration of the vegetation band.

This argument applies in the same way for nonlocal plant dispersal, enabling moving patterns for both large and small widths of dispersal kernel (Fig. 1). However when the kernel has low amplitude and wide span (small $\eta$ in (3)), a new solution type becomes possible, in which sharp peaks of vegetation are separated by regions with very low vegetation. Then dispersal only increases the vegetation density between the bands by a relatively small amount, and approximately uniformly, because of the small value of $\eta$. Typically this will not be sufficient to move the solution out of $\mathcal{B}_{\mathrm{no-veg}}$. Indeed, this nonlocal dispersal actually leads to a transition of the solution from $\mathcal{B}_{\textrm{veg}}$ to $\mathcal{B}_{\mathrm{no-veg}}$ on both sides of the vegetation spike (Fig. 9b, bottom). Thus dispersal does not induce further increase in vegetation density between the bands, and the solution remains fixed in space.

To conclude, we discuss some future research directions suggested by our work. Prominent amongst these is whether our results depend in any fundamental way on the shape of the dispersal kernel. We have restricted attention to the Laplace kernel (3), but this is for purely mathematical reasons: for this kernel, the travelling wave equations can be reduced to a system of ordinary differential equations. For more general kernels, a corresponding reduction is not possible and one must work with integrodifferential travelling wave equations. We have not been able to generalise our mathematical results, but investigation of these travelling wave integrodifferential equations is an important target for future work. In particular, it would be interesting to study the possibility of almost stationary patterns for “fat” dispersal kernels (with algebraic rather than exponential decay to zero)—such kernels are realistic for many plant species (Bullock et al. 2017; Kot et al. 1996). They yield significantly different behaviours in the context of travelling wave fronts (Liu and Kot 2019), but to our knowledge there has not been any work on semi-arid vegetation patterns using fat-tailed dispersal kernels.

Another natural direction for future work is to consider whether long-range dispersal can lead to almost-stationary patterns in other models of banded vegetation. The model of Rietkerk et al. (2002) differs from the Klausmeier model (1) in having separate variables for water above ground and in the soil. This was the first model of semi-arid vegetation into which long-range plant dispersal was introduced (Pueyo et al. 2008), but these authors restricted attention to flat ground. The possibility of almost-stationary patterns on a slope is an important topic for future work. Another widely used model for semi-arid vegetation is that of Gilad et al. (2007), which includes an integral term to represent the dependence of the growth rate of a plant on soil water levels in a wide area around it, because of the extensive root systems that characterise semi-arid vegetation. To our knowledge, this model has never been considered with a separate integral term for long-range dispersal, and the possibility of almost stationary patterns in such an extended model is another important topic for future work. Similarly, nonlocal seed dispersal terms could in the future be included in kernel-based models for dryland vegetation patterns. In models such as those by Borgogno et al. (2009), Escaff et al. (2015), Lefever and Lejeune (1997), Martinez-Garcia et al. (2013), plant-to-plant interactions are represented by nonlocal kernel terms. Thus, this type of model is ideally suited for the additional inclusion of a kernel-based seed dispersal term in future work.

A third area for future work is to investigate the form of spatial patterns when $d>d^*$. As we have discussed, our layered solution breaks down at $d=d^*$ because there is no solution of the outer equations (14) subject to the boundary condition (21a). However, numerical continuation suggests that almost stationary patterns continue to exist for $d>d^*$. Moreover there is no apparent change in pattern form as *d* increases through $d^*$. We have been unable to determine an appropriate solution structure for $d>d^*$, and this remains a pressing issue for future research.
